# Web-based cognitive behavioural therapy (W-CBT) for diabetes patients with co-morbid depression: Design of a randomised controlled trial

**DOI:** 10.1186/1471-244X-8-9

**Published:** 2008-02-19

**Authors:** Kim MP van Bastelaar, Frans Pouwer, Pim Cuijpers, Jos WR Twisk, Frank J Snoek

**Affiliations:** 1 Department of Medical Psychology, VU University Medical Centre, The Netherlands; 2 EMGO Institute, VU University Medical Centre, The Netherlands; 3 Department of Clinical Psychology, VU University Medical Centre, The Netherlands; 4 Department of Clinical Epidemiology and Biostatistics, VU University Medical Centre, The Netherlands; 5 VU University Medical Centre, Department of Medical Psychology, Diabetes Psychology Research Group, Van der Boechorststraat 7, 1081 BT Amsterdam, The Netherlands

## Abstract

**Background:**

Depression is common among people with diabetes, negatively affecting quality of life, treatment adherence and diabetes outcomes. In routine clinical care, diabetes patients have limited access to mental health services and depression therefore often remains untreated. Web-based therapy could potentially be an effective way to improve the reach of psychological care for diabetes patients, at relatively low costs. This study seeks to test the effectiveness of a web-based self-help depression programme for people with diabetes and co-morbid depression.

**Methods/Design:**

The effectiveness of a web-based self-help course for adults with diabetes with co-morbid depression will be tested in a randomised trial, using a wait-list controlled design. The intervention consists of an 8-week, moderated self-help course that is tailored to the needs of persons living with diabetes and is offered on an individual basis. Participants receive feedback on their homework assignments by e-mail from their coach. We aim to include 286 patients (143/143), as power analyses showed that this number is needed to detect an effect size of 0.35, with measurements at baseline, directly after completing the web-based intervention and at 1, 3, 4 and 6 months follow-up. Patients in the control condition are placed on a waiting list, and follow the course 12 weeks after randomisation.

Primary outcomes are depressive symptoms and diabetes-specific emotional distress. Secondary outcomes are satisfaction with the course, perceived health status, self-care behaviours, glycaemic control, and days in bed/absence from work. Questionnaires are administered via the Internet.

**Discussion:**

The intervention being trialled is expected to help improve mood and reduce diabetes-specific emotional distress in diabetes patients with depression, with subsequent beneficial effects on diabetes self-care and glycaemic outcomes. When proven efficacious, the intervention could be disseminated to reach large groups of patients with diabetes and concurrent depressive symptoms.

**Trial registration:**

Current Controlled Trials ISRCTN24874457

## Background

Depression has been shown two times more prevalent among persons with type 1 or type 2 diabetes, compared to the general population [1]. Approximately 10% of the adult diabetes patient population suffers from major depressive disorder and another 10% from minor depression [1]. Data also suggest depression to be more persistent and recurrent in people with diabetes [2]. The costs of depression in diabetes are known to be high, not only in terms of suffering and reduced quality of life, but also in view of adverse medical outcomes (e.g. hyperglycaemia, diabetes complications), and societal and economic costs [3,4].

Based on evidence to date, psychological therapy (particularly cognitive behaviour therapy, CBT) is the treatment of choice for depression [5]. Only a few randomised controlled studies have been conducted to test the efficacy of anti-depressant therapies in persons with diabetes and co morbid depression [6-8].

Not surprisingly, depressive symptoms are likely to co-occur with high levels of diabetes-related emotional distress, as was confirmed in a recent international study [9]. While the optimal treatment for depression in diabetes is still being sought [10], there is good reason to assume that the efficacy of anti-depressant psychotherapy in diabetes can be enhanced when specific issues associated with the burden of living with this chronic disease are adequately addressed [11].

We recently conducted a study to test the effectiveness of a group Cognitive Behaviour Therapy (CBT) developed for patients in prolonged poor glycaemic control in a RCT, and found CBT to be most effective in patients who entered the study with elevated depression scores [12,13].

Offering diabetes-specific CBT to diabetes patients with depression should thus be able to improve psychological health, self-management behaviours and subsequent medical outcomes. However, access to psychological services is limited, both in primary and secondary routine diabetes care [10]. As pointed out by Glasgow et al [14], we need to consider ways to enhance dissemination of effective psychological interventions to persons with diabetes. In this context, we believe use of modern interactive technology, such as internet-based therapy should be considered. More so, since web-based psychological interventions have shown their utility across a range of problem areas and are likely to be cost-effective [15,16].

Web-based cognitive behaviour therapy (W-CBT) is recognized as an effective treatment option for depression and appeared to be well appreciated by patients [15,17-20]. In The Netherlands, we have seen an increase of internet-use in the past years, with about 83% of the households currently being "online" [21]. Internet-based therapy therefore has great potential to reach large groups of diabetes patients with co-morbid depression. To our knowledge, we would be the first to test the effectiveness of diabetes-specific on-line CBT for depression in persons with diabetes.

### Aims of the Trial

This study aims to test the effectiveness and appreciation of web-based cognitive behavioural therapy (W-CBT) for adult diabetes patients with depression in a randomized controlled trial. The experimental condition will be compared with a waiting-list control group. It is hypothesized, that W-CBT will be well-received by the participants and will appear to be significantly more effective than the control condition in reducing levels of depressed mood and diabetes-specific emotional distress, with subsequent positive effects on self-management behaviours and glycaemic control on the longer term.

## Methods/Design

### Study Design

**Figure 1:**
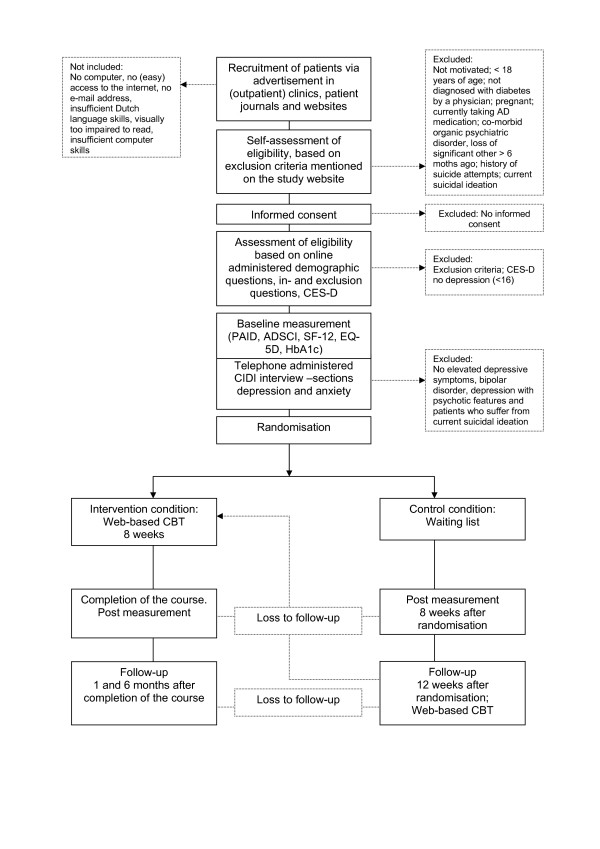
**Flow chart of participants**. AD: anti-depressives; CES-D: Center for Epidemiologic Studies Depression scale; PAID: Problem Areas In Diabetes scale; ADSCI: Diabetes Self-care Inventory; SF-12: Short-Form-12; EQ-5D: Euroqol 5D; CBT: Cognitive behavioural therapy.

We chose a two-arm randomised controlled trial (RCT) design; including 286 patients (143/143) (see power calculation). Measurements are scheduled at six points in time in the intervention group: at baseline, directly after completing the web-based intervention, and at 1, 3, 4 and 6 months follow-up. In the control group the same six measurements are scheduled with two additional measurements at 8 and 12 weeks after randomization (see flow chart, Figure 1).

### Study procedures

The recruitment and screening procedure has been developed, so that patients interested in joining the study can visit the research website. There, the patient will be invited to read the study information and provide written informed consent. After receipt of the consent form, the patient will receive a password per e-mail allowing him/her to log in and complete the online screening questions. For the baseline measurement, patients are invited to fill out a booklet of questionnaires on the Internet, -which will take approximately 30 minutes-containing demographic data, in – and exclusion criteria, and depressive symptoms (Center for Epidemiological Studies Depression scale (CES-D) >16) [22]. If a patient does not qualify, this is explained and the patient is advised to contact his/her GP for further advice. If eligible, the patient will be interviewed by telephone using the Composite International Diagnostic Interview (CIDI). Data from this CIDI interview will be used to specify type of mood disorder (DSM-IV). Those with bipolar disorder, depression with psychotic features and patients who suffer from current suicidal ideation will be excluded. After eligibility is confirmed the patient is enrolled in the study.

### Treatment allocation

Randomisation by computer will assign individual patients to either the experimental or control condition.

### Recruitment

The on-line depression course will be delivered through a website. The study will be advertised in the Netherlands and Flanders (Dutch speaking part of Belgium) in clinics (e.g. hospitals, GP's, pharmacy's, rehabilitation centres) and through various media (e.g. patient journals, specialist journals, websites, e-mails, flyers, newspapers etc.). It will be advertised as a web-based course for persons with diabetes (type 1 or type 2) to help improve a depressed mood. Patient information on the study will be available on the research website. Inclusion and exclusion criteria will be stated clearly on this website.

### Study population

The source population consists of adult diabetes patients with co-morbid depression. Blinding of subjects will -due to ethical reasons- not be carried out.

Inclusion criteria are: ≥18 years of age; having type 1 or type 2 diabetes (diagnosed > 3 months prior to study by a physician); having a depressed mood, as indicated by a score of 16 or higher on the Center for Epidemiological Studies Depression scale (CES-D); depression diagnosed by the CIDI-interview; having access to the Internet at home and having an e-mail address.

Exclusion criteria are: not having easy access to the Internet, reading problems (e.g. due to insufficient Dutch language skills, visual impairments or illiteracy); currently taking anti-depressant medication; a history of suicide attempt(s); current suicidal ideation (measured with the CIDI); bipolar disorder (CIDI); co-morbid organic psychiatric disorder (CIDI); loss of significant other < previous 6 months; or pregnancy.

### Description of interventions

#### Intervention group

Patient enrolment will occur individually on a continuous basis, i.e. at any point in time during the study period. The number of included participants is not limited by physical capacity other than the number of coaches available. After inclusion criteria are met and the patient has returned the informed consent s/he will be randomly assigned to the intervention or the control group.

Patients assigned to the intervention group will receive a password per e-mail with which they can log in to the course. They will be instructed (and reminded per e-mail) to complete one session per week, during 8 consecutive weeks.

Coaches will send e-mails to participants as a way to encourage them to continue their efforts. Such reinforcement should help to keep patients on track with the course and lower the risk of drop-out. They also provide the participants with feedback on their homework assignments. It is clearly stated that coaches will not give advice on personal issues.

#### Control group

Control group patients will receive an e-mail in which they are informed of the randomisation result, which means they will start the course after 12 weeks. To reduce the risk of loss of interest; participants placed on the waiting-list will automatically receive weekly protocollised e-mails with motivating, positive feedback to enforce them to remain involved in the study.

After eight weeks they are invited per e-mail to fill in the questionnaires again. Twelve weeks after randomization patients will undergo a second screening (using the CES-D-score ≥ 16 as a first screener for depression and additionally the CIDI interview) to confirm depression. If still eligible, the patient is invited to follow the course. Patients, who do not qualify, i.e. show (spontaneous) remission, are excluded at this point. Patients who developed suicidal ideation during the 12 weeks waiting period, are offered the opportunity to have an appointment with a diabetes psychologist of the VU University Medical Centre or a colleague nearby in case the distance from the participant's home to the VUMC should be problematic.

The effectiveness of the intervention is determined directly after completing the web-based intervention up till 6 months follow-up (see Consort flow chart in Figure 1).

If the depression has deteriorated at the end of the course, as indicated by the scores of the CES-D, a consultation with a diabetes psychologist at the VU Medical Centre is offered to the patient. In agreement with their General Practitioner a decision will be made about further treatment or hospitalization.

### Intervention development and trial design

The Dutch version of the manual-based self-help course named 'Coping with Depression' ('In de put, uit de put') has been adapted as web-based intervention ('Kleur je Leven') [23,24]. This intervention has been adjusted further by our team to fit the needs of patients with diabetes ('Diabetergestemd'). Based on our research, clinical experience and input from an expert panel of diabetes patients, the following topics were incorporated: managing 'poor' test results, uncertainty about blood glucose fluctuations and negative emotions, communication with health care professionals, talking about diabetes with others, the burden of daily self-management, and coping with diabetes-related worries (e.g. about hypoglycaemia and late complications). The course consists of 8 consecutive weekly lessons. The course will provide information, practice examples, exercises/self-tests and homework assignments. The web-based course is delivered on an individual basis. Patients weekly receive (protocollized) feedback on their homework assignments from their coaches by e-mail. The coaches are psychologists and residents in clinical psychology from the department of Medical Psychology of the VU medical centre, supervised by the research team. An internet-based group forum, which will be moderated by our team, is offered to participants to give them the opportunity to share experiences, provide support and discuss issues related to depression and diabetes [25]. In the forum, specific treatment issues (e.g. related to oral medication or insulin) may not apply to all participants, but this should not be problematic. Rather, the mix of disease types and experiences can serve to illustrate the common features of diabetes and depression (thoughts, emotions and behaviors), while respecting individual differences.

It will be clearly stated that during the course of the study, all participants are allowed to make use of additional mental health care services if they feel a need to do so.

### Outcome Assessment

#### Primary outcome measures

Primary outcome measure is the level of depression, measured by the online administered self-reported depression questionnaire Center for Epidemiological Studies Depression scale (CES-D) [26] at baseline, directly after completion of the course and at one and six months follow-up.

#### Secondary outcome measures

Secondary outcome measures are: diabetes-specific emotional distress (PAID) [27], satisfaction with the course (self-developed questions), perceived health status (EQ5D and SF-12) [28], diabetes self-care behaviours (Amsterdam Diabetes Self Care Inventory), self-reported episodes of hypoglycaemia and glycaemic control (HbA_1c_) retrieved from the patients' medical charts via their physician.

#### Covariates

Additionally, information on potentially confounding factors and effect modifiers will be collected by means of self-report: socio-demographic data (age, gender, marital status, highest level of completed education, and current occupation), lifestyle issues (smoking, body mass index (BMI), substance/alcohol abuse), data on diabetes (type of diabetes, duration, treatment regimen, co-morbidity) and additional mental health care consumption.

### Statistical Analyses

Analyses will be conducted according to the intention to treat principle. The data will be presented as categorical and continuous variables. Baseline characteristics will be compared for the intervention and waiting-list group using Student *t*-tests and χ^2^-tests. Analysis of variance (ANOVA) will be conducted with the CES-D (depression) as dependent variable, with two independent variables: time (within-subject) and group (between-subject). The same analysis will be performed with the PAID score (diabetes-related emotional distress) as dependent variable. Two analyses will be conducted. Firstly, the direct effect of the intervention will be determined. ANCOVA's will be performed in order to test whether both groups have different scores on the CES-D, at different points in time, with correction for potential confounders at baseline. Secondly, pre-post measurements will be conducted of all participants who have received the intervention. This will be analysed by means of a longitudinal regression analysis, which will be performed using Generalized Estimating Equations (GEE) [29], taking into account the correlational nature of repeated measures data within subjects, and securing minimal loss of patients due to incomplete data. Clinical effectiveness will be calculated by the amount of people who after completion of the course have a score on the CES-D of <16. The effect size will be calculated using Cohen's *d *[30].

### Power calculation

The sample size was calculated using STATA, based on clinically relevant differences. Effect Sizes (ES) are calculated as the difference between the control group and the intervention group after the intervention, divided by the pooled standard deviation [30]. With 100/100 patients the study is powered to detect an ES of 0.35 (one-sided), with a power of 80% (α = 0.05). Based on a meta-analysis [23], we may expect an ES of 0.5 to 0.6 [18]. We anticipate 30% non-completion/exclusion, which will be compensated by over sampling (n = 286; 143 in each group).

## Discussion

### Strengths and Limitations

To the best of our knowledge, this randomised controlled trial will be the first to test the effects of a web-based CBT self-help course for people with diabetes and co-morbid depression. The intervention builds on an existing course that has shown to be feasible and effective to help improve mood in the general population [31]. Our trial is sufficiently powered to allow for conclusions as to its short-term effectiveness (6 months). In view of the "lenient" inclusion and exclusion criteria we will apply, the study is expected to have high external validity.

An obvious limitation of this trial is the fact that only people with access to the Internet and sufficient computer skills can be included. Overall, an estimated 82% of the Dutch households are online in 2007, but access to Internet among the elderly is much lower [21] and as a consequence this group may therefore be under represented in our study.

Inclusion and exclusion criteria are clearly stated on the website. This has the potential disadvantage that people can pretend to have certain characteristics in order to qualify or they can deny having "exclusion criteria". However, this response tendency may be diminished by the fact that patients are being made aware that we inform their GP's of their participation in the study and that we will be in contact with their treating physician concerning their HbA1c results.

As the post-treatment assessment will be done directly after treatment and up till 6 months follow-up, no conclusions can be drawn about the long-term effects of the intervention. Therefore, after 6 months, patients will be asked to give permission for supplemental follow-up measurements at one and two years after completion of the course. These results will be analysed at a later stage and are not part of this project.

Although web-based administration of questionnaires is widely used, we should remind ourselves that the psychometric properties of internet-administered questionnaires may not be equal to those of paper-and-pencil versions [32]. On the other hand, in an earlier study we have found that the paper-and-pencil and the computerised versions of a short psychological well-being questionnaire appeared to be equivalent [33]. The major difference appears to be that respondents are more honest and revealing when filling out via the Internet, resulting in a relatively higher reported level of symptomatology. We have therefore chosen to confirm or decline the diagnosis of depression by a telephone administered diagnostic interview (CIDI). Our study will show if such diagnostic procedure is required in future projects applying W-CBT in order to reduce the risk of 'false positives'.

Also the psychometric properties of the following on-line questionnaires will be examined and compared to the paper-and-pencil versions: the PAID, SF-12 and the ADSCI.

We hypothesise that improved mood will be associated with improved diabetes self-management and we will test whether W-CBT has favourable effects on self-reported diabetes self-care behaviours, using the Amsterdam Diabetes Self-care Inventory (ADSCI) that was developed by our team and used in previous trials [12,13,34]. The psychometric properties of the Amsterdam Diabetes Self Care Inventory (ADSCI) have not yet been published, but a report on the validity and reliability of the scale is planned based on data on file.

Ethical and practical considerations have led us to decide that all participants are allowed to use additional mental health care (MHC) services during the trial. Usage of additional services therefore is a potential confounder that will be measured and taken into account in the statistical analyses.

Drop-out is often a problem in web-based therapy trials. In adapting the generic coping with depression course to the needs of people with diabetes, we expect to reduce drop-out. In an attempt to further reduce the attrition rate, support e-mails will be send out to participants by their coach, to encourage them to stay involved in the course. Likewise, those in the waiting list condition will receive e-mails aimed to maintain contact and encourage them to participate.

The study protocol was approved by the VU University Medical Centre ethics committee, which is certified by the Central Committee on Research involving Human Subjects in the Netherlands.

### Future implementation

If this intervention proves to be effective in reducing depression and shows to be well appreciated by diabetes patients, further dissemination of the intervention is anticipated. An implementation and dissemination plan is under development.

This intervention could also be adapted to suit the needs of people who suffer from chronic diseases other than diabetes.

## Competing interests

The author(s) declare that they have no competing interests.

## Authors' contributions

KB constructed the design of the study and drafted the manuscript.

FP constructed the design of the study and revised the manuscript.

PC participated in the design of the study and revised the manuscript.

JT helped to develop the statistical analyses and reviewed the manuscript.

FS developed the study, constructed the design and revised the manuscript.

All authors read and approved the final manuscript.

## Pre-publication history

The pre-publication history for this paper can be accessed here:


